# The impact of gut bacteria producing long chain homologs of vitamin K_2_ on colorectal carcinogenesis

**DOI:** 10.1186/s12935-023-03114-2

**Published:** 2023-11-10

**Authors:** Joanna Smajdor, Katarzyna Jedlińska, Radosław Porada, Anna Górska-Ratusznik, Aleksandra Policht, Małgorzata Śróttek, Grażyna Więcek, Bogusław Baś, Magdalena Strus

**Affiliations:** 1 Department of Analytical Chemistry and Biochemistry, Faculty of Materials Science and Ceramics, AGH University of Science and Technology, Al. Mickiewicza, Kraków, 30-059 Poland; 2 Department of Analytical Chemistry, Faculty of Chemistry, Jagiellonian University, Gronostajowa 2, Kraków, 30- 387 Poland; 3 Sieć Badawcza Łukasiewicz—Krakowski Instytut Technologiczny, ul. Zakopiańska 73, Cracow, 30-418 Poland; 4 Chair of Microbiology, Jagiellonian University Medical College, Czysta 18, Krakow, 31-121 Poland

**Keywords:** Colorectal cancer, Carcinogenesis, Microbiome, Vitamin K_2_, K_2_-MK7

## Abstract

Colorectal cancer (CRC) is one of the foremost causes of cancer-related deaths. Lately, a close connection between the course of CRC and the intestinal microbiota has been revealed. Vitamin K_2_ (VK_2_) is a bacterially derived compound that plays a crucial role in the human body. Its significant anti-cancer properties may result, inter alia, from a quinone ring possessing a specific chemical structure found in many chemotherapeutics. VK_2_ can be supplied to our body exogenously, i.e., through dietary supplements or fermented food (e.g., yellow cheese, fermented soybeans -Natto), and endogenously, i.e., through the production of bacteria that constantly colonize the human microbiome of the large intestine.

This paper focuses on endogenous K_2_ synthesized by the most active members of the human gut microbiome. This analysis tested 86 intestinally derived bacterial strains, among which the largest VK_2_ producers (Lactobacillus, Bifidobacterium, Bacillus) were selected. Moreover, based on the chosen VK_2_-MK4 homolog, the potential of VK_2_ penetration into Caco-2 cells in an aqueous environment without the coexistence of fats, pancreatic enzymes, or bile salts has been displayed. The influence of three VK_2_ homologs: VK_2_-MK4, VK_2_-MK7 and VK_2_-MK9 on apoptosis and necrosis of Caco-2 cells was tested proving the lack of their harmful effects on the tested cells. Moreover, the unique role of long-chain homologs (VK_2_-MK9 and VK_2_-MK7) in inhibiting the secretion of pro-inflammatory cytokines such as IL-8 (for Caco-2 tissue) and IL-6 and TNFα (for RAW 264.7) has been documented.

## Introduction

Colorectal cancer (CRC) is the third most common cancer and the leading cause of death from gastrointestinal malignancy in the United States [1]. Globally, it is the second most malignant neoplasm in women after breast cancer and the third in men, after lung cancer and prostate cancer. Based on current epidemiological data, CRC is more prevalent in Europe, North America, and Australia than in Asia [2, 3]. The main risk factors for developing CRC are genetic background, food choices, age, environment, lifestyle, physical activity, and obesity. Moreover, chronic gut diseases can prolong and increase gastrointestinal tract inflammation, which significantly increases the risk of CRC [4].

Numerous reports deliver solid scientific evidence for the crucial role of the intestinal microbiota in maintaining good health and disease prevention [5–8]. It is estimated that the total number of bacteria can reach up to 10^14^ cells/g of stool in the large intestinal lumen. Such a vast mass of gut bacteria (about 2 kg in an adult human intestine) serves as a separate organ in our body responsible for intestinal barrier integrity and proper functioning of the immune system. Through the release of small peptides, volatile fatty acids, enzymes, and reactive oxygen species, the gut microbiota can modify many life processes in the intestines, including carcinogenesis. The human intestinal microbiota is also responsible for synthesizing specific B and K group vitamins considered precursors and catalysts for many chemical processes and metabolic pathways [9–12]. Many in vitro and in vivo studies have supported the correlation between a properly working human gut microbiota and CRC prevention and treatment [13–15].

We have observed a significant increase in publications on K vitamins’ properties, occurrence, and role in life processes in recent years [16–22]. There are three basic types of vitamin K: K_1_ (phylloquinone), K_2_ (menaquinone), and K_3_ (menadione). Phylloquinone is found in vegetables, primarily the dark green parts. In contrast, menaquinone is synthesized by specific bacteria species in the human gut and during bacterial fermentation of certain food products. Menadione does not occur naturally but can be administered as a synthesized drug to patients.

VK_2_ is a fat-soluble vitamin that, through food fermentation, is synthesized by some bacterial species in the human digestive tract and beyond. The ring structure of 2-methyl-1,4-naphthoquinone is characteristic of all vitamin K_2_ homologs, but they differ by the side chain length composed of unsaturated isoprenoid units (which may count from n = 1 to n = 13, VK_2_-MKn). In recent years, much interest has been placed on VK_2_ impact on the human body and the methods of sourcing it from natural products. Several studies have demonstrated that some vitamin K_2_ homologs regulate the calcium equilibrium by binding and transporting calcium to the skeletal system, preventing blood vessel calcification [23–25].

Because the quinone ring’s chemical structure is utilized in many chemotherapeutics, different VK_2_ homologs are considered prophylaxis and neoplastic disorder treatment forms. Thus, ongoing trials are trying to determine the biological function of vitamin K_2_ in carcinogenesis inhibition [26, 27]. It has been shown that colon cancer can be inhibited in mouse cells in vitro by vitamin K and also that vitamin K acts as an anti-cancer agent in forming colon cancer [28]. In vitro studies conducted by Dasari’s [29] team present potential anticancer effects of vitamin K in castration-resistant prostate cancer. Moreover, in mice they show inhibitory effects on androgen-dependent and independent tumor growth. [30, 31].

Nowadays, vitamin K_2_ deficits are replenished by oral dietary supplements containing mainly the synthetic K_2_-MK7 homolog (the most stable form compared to other VK_2_ varieties). Nevertheless, it is not entirely the right course of action as a single high dose administration may only be partially absorbed [32–34]. Indirect VK_2_ supplementation (typical for the human intestinal microbiota) through bacteria producing it is worth considering. Current literature indicates that bacterially synthesized VK_2_ plays an essential role in meeting nutritional vitamin K demands, wheres its deficiency leads to clinically significant coagulopathy. The K_2_-MK7 homolog is detected in large amounts in traditional Japanese Nattō, and Korean Cheonggukjang – both these products are derived from soybeans fermented by *Bacillus subtilis Natto* strains [35–37]. However, *Bacillus subtilis* is not a representative species of the microbiota of the human gastrointestinal tract; rather it is a planktonic microorganism only temporarily colonizing the intestinal epithelium. Additionally, the taste, smell, and texture of Nattō are generally tolerable solely to residents of the Far East (Japan, Korea, China).

These two reasons lead to an ongoing search for other intestinal probiotic strains, which would not only be able to colonize human intestinal epithelium quickly and for an extended period of time, but also have the natural vitamin K_2_ synthesis ability in amounts comparable to Nattō.

Thus, the research sought to investigate which cultivated bacteria species inhabiting the human microbiome can be regarded as potent vitamin K_2_ producers. We strived to compare the amount of VK_2_ produced in the culture medium by different strains of bacteria belonging to the same species while maintaining a comparable number of bacteria of each strain at a similar level of 10^9^ CFU/g (colony-forming unit per gram). We likewise examined the effect of selected synthetic VK_2_: K_2_-MK4, K_2_-MK7, and K_2_-MK9 homologs on apoptosis, necrosis, and the secretion of pro-inflammatory cytokines released by human Caco-2 intestinal epithelial cells and RAW 264.7 mouse macrophage cells. In addition, we attempted to see whether the chosen VK_2_-MK4 homolog can penetrate deeply into the host’s eukaryotic cells in the aquatic environment, despite the absence of fats, bile salts, or pancreatic enzymes.

## Materials and methods

### Bacterial strains and culture conditions

The bacterial strains used in this study were part of the collection of Jagiellonian University Medical College strains isolated from the healthy human gastrointestinal tract during research conducted in accordance with the original protocols PB-DM/SBK-NEC-01/11 and KBET/236/B/2002. Depending on bacterial species, different culture mediums were used, such as TSB Broth (Becton Dickinson) for *Bacillus, Escherichia, Klebsiella, Enterococcus, Staphylococcus, Enterobacter, Hafnia* and *Pseudomonas*; MRS Broth (DeMan-Rogosa-Sharpe) (Oxoid, UK) for *Lactobacillus*; Schaedler Broth (Sigma-Aldrich) for *Clostridium*; and TOS Broth (Sigma-Aldrich) for *Bifidobacterium* genus. The inoculum was prepared by multiplication of 24- or 48-h old cultures and suspending the material in 50 mL of sterile culture medium in Erlenmeyer flasks to get a final concentration of 10^7^ CFU/g. Anaerobic and microaerophilic bacterial genus, such as *Lactobacillus, Bifidobacterium* and *Clostridium* were cultivated maintaining anaerobic conditions by using anaerobic chambers (GENbox anaer, bioMerieux SA, France) and CO_2_ generators (BD GasPak™ EZ Container System, BD Diagnostics, USA). In the case of other species, the cultivation process was carried out under aerobic conditions. Flasks prepared with bacterial culture were statically incubated at 37 °C for the time of 24, 48, 72 and 96 h, without access to light in order to avoid the process of VK_2_ degradation.

### Preparation of the bacterial supernatant sample for voltammetric measurements

For each measurement day 3 flasks with each bacterial strain culture were prepared. Flasks were shaken in order to homogenize the cultivation. Next, the content of the flasks was transferred to sterile centrifuge tubes, one for each culture. Tubes were centrifuged for 5 min with a speed of 10 000 rpm. Supernatant obtained in this way was filtered using the PES syringe filter with the pore size of 0.22 μm (Biosens). The volume of filtered supernatant was about 20 mL. 50 µl of supernatant was used for voltammetric measurements. All samples were protected from light during the VK_2_ determination.

### Analysis of vitamin K_2_ content in bacterial supernatants

The total VK_2_ content (sum of homologs) in all supernatants was determined by Differential Pulse Adsorptive Stripping Voltammetry (DP AdSV) using the measurement procedure described in the literature by our team [38, 39]. This method meets all the criteria of a screening method. For every tested strain, three separate sets of cultures were prepared, for which the analysis of VK_2_ content in supernatant was performed after 24, 48, 72 and 96 h of cultivation. Thus, 12 values of VK_2_ concentration were obtained per each strain.

Voltammetric measurements were performed on the M20 multipurpose electrochemical analyzer coupled with the M164 electrode stand (both mtm-anko, Poland) and equipped with the EAPro 1.0 software. All measurements were performed using the three-electrode cell, including the Controlled Growth Mercury Drop Electrode (CGMDE, 1.2 mm^2^) as a working electrode, the double junction silver chloride reference electrode (Ag/AgCl/3 M KCl/2.5 M KNO_3_) and a platinum wire as the auxiliary electrode. Every week, a fresh stock standard solution of vitamin K_2_ (500 mg/L) was prepared by dissolving the dry VK_2_ in methyl alcohol, and then was stored in an amber volumetric flask in the freezer at -20 °C. All diluted solutions of VK_2_ were prepared from the stock solution shortly before the measurements. A mixture of 70% (v:v) of methanol and 0.30 M acetate buffer (pH 3.8) was used as the supporting electrolyte. To ensure the proper electrolytic conductivity 0.1225 ± 0.0005 g of sodium perchlorate was added to 5 mL of supporting electrolyte. Analyzed solution was deoxygenated with argon for 3–5 min before measurements.

DP AdSV voltammograms were recorded in the potential range from − 0.04 to − 0.4 V in both, cathodic and anodic direction, under optimal measurement conditions: potential step E_s_ = 2 mV, pulse amplitude dE = 30 mV, and pulse period t_imp_ = (t_w_ + t_s_) = 10 ms; in each case it was assumed that t_w_ = t_s_ (waiting time = current sampling time). Accumulation step was performed before both, cathodic (accumulation potential E_acc1_ = − 0.04 V, accumulation time t_acc1_ = 1 s) and anodic scans (E_acc2_ = − 0.4 V, t_acc2_ = 30 s). The use of these two stages of VK_2_ accumulation on the CGMDE surface allows obtaining a sensitivity unattainable for other methods of instrumental analysis. To maintain a satisfactory repeatability and reproducibility, the measurements were carried out in an air-conditioned room at temperature of 22 ± 1 °C. Quartz measuring cells with test solutions were protected against light with an aluminum foil.

### Preparation of the Caco-2 and the RAW 264.7 cell lines

Human colon adenocarcinoma cell line Caco-2 was obtained from the Sigma-Aldrich (LOT 17H003). Cells were grown in culture medium – Dulbecco’s modified Eagle medium (DMEM, Sigma-Aldrich) with 4.5 g glucose per liter, supplemented with 1% (v/v) nonessential amino acids, 0.2 mM L-glutamine, 1% penicillin - streptomycin - neomycin solution and 10% (v/v) fetal calf serum (FCS) (all regents from Sigma-Aldrich), at 37 °C in a humidified atmosphere of 5% CO2 in the air. The cells were cultivated in 75 cm^2^ tissue culture flasks and routine renewal of cell stocks was carried out twice a week by removing cells with a solution containing 0.25% (w/v) trypsin and 0.02% (w/v) EDTA in calcium-free and magnesium-free phosphate-buffered saline solution (PBS), pH 7.4 (both from Sigma-Aldrich).

RAW 264.7 mouse macrophage cell line was obtained from Sigma-Aldrich (LOT 17K027). Cells were grown in Dulbecco’s modified Eagle medium (DMEM, Sigma-Aldrich) with 3.7 g sodium bicarbonate per liter, 2 mM L-glutamine, 1% penicillin - streptomycin - neomycin solution and 10% (v/v) fetal calf serum (FCS) (Sigma-Aldrich) at 37 °C in an atmosphere of 7.5% CO_2_. The cells were cultivated in 75 cm^2^ tissue culture flasks and routine renewal of cell stocks was carried twice a week. After reaching the confluency of 80–90%, the culture medium was removed, and cells were used for further experiments.

All cell lines were routinely tested for mycoplasma by Polymerase Chain Reaction (PCR).

#### Influence of vitamin K_2_ homologs on apoptosis and necrosis of Caco-2 cells

Caco-2 cells cultivated as described in Sect. 2.4 were seeded in 24-well plates (1·10^5^ cells/well) in the medium containing 5% FBS (Fetal Bovine Serum). When the cells reached monolayer with 85% confluence, they were cultivated with different concentrations of three VK_2_ homologs: K_2_-MK4 and K_2_-MK7 (both Sigma-Aldrich) and K_2_-MK9 (Cayman Chemical) in order to describe the influence of VK_2_ homologs on apoptosis and necrosis of Caco-2 cells. The control group consisted of Caco-2 cells was treated with cell medium only. The positive control group consisted of Caco-2 cells was cultivated with 2 µM staurosporine. After 24, 48 and 72 h, the cells were washed twice with PBS. Double staining with 5 µg/mL Hoechst 33,342 dye (Life Technologies, USA) and Annexin-V-FLUOS staining kit (Roche Diagnostics GmbH, Germany), containing annexin-V-fluorescein and propidium iodide (PI) was performed to quantify the number of apoptotic and necrotic cells in culture on the basis of scoring cell nucleus. A fluorescence microscope, BX51 (Olympus Europe, Germany), with the appropriate filters was used to count the number of apoptotic (Annexin V-positive cells) and necrotic (stained with propidium iodide) cells in five random fields of view using the 20× microscope objective. These numbers were compared to the total number of cells (based on Hoechst staining) and the results are presented in percentages. During the whole sample preparation process and measurements, VK_2_ standards and samples were preserved from the light.

#### Influence of vitamin K_2_ homologs on cytokines

In order to describe the influence of VK_2_ on the secretion of cytokines Caco-2 cells cultivated as described in Sect. 2.4 were seeded in 24-well plates (1·10^5^ cells/well) in medium containing 5% FBS. When the cells reached monolayer with 85% confluence, they were cultivated during the period of 24 h with different concentrations of three VK_2_ homologs: K_2_-MK4, K_2_-MK7, K_2_-MK9. The control group consisted of Caco-2 cells was treated with cell medium only. After the allotted time, the supernatants of Caco-2 culture were collected directly on 96 well sterile plate and stored frozen. Cytokine concentrations in Caco-2 culture supernatants were measured using ELISA method, accordant to the manufacturer’s instructions (Invitrogen). In this study, the pro- and anti-inflammatory cytokine profile (Tumor Necrosis Factor α (TNFα), Interleukin 6 (IL-6), Interleukin 8 (IL-8) and Interleukin 10 (IL-10)) was tested. Each measurement was repeated three times. During the whole sample preparation process and measurements, VK_2_ standards and samples were preserved from the light.

RAW 264.7 cells cultivated as described in Sect. 2.4 were seeded in 24-well plates (1·10^5^ cells/well) in cell culture medium. When the cells reached monolayer with 85% confluence, they were cultivated for 24 h with different concentrations of vitamin K_2_-MK4, K_2_-MK7, K_2_-MK9 and with addition of 100 ng/mL of lipopolysaccharide (LPS) (Sigma-Aldrich). After the allotted time, the supernatants of RAW 264.7 culture were collected directly on 96 well sterile plate and stored frozen before measurements. Cytokine concentrations in RAW 264.7 culture supernatants were measured using ELISA method, in accordance with the manufacturer’s instructions (Invitrogen). Due to specification of RAW 264.7 tissue, the pro-inflammatory cytokine profile (TNFα and IL-6) was tested. Each measurement was repeated three times. During the whole sample preparation process and measurements, vitamin K_2_ standards and samples were preserved from the light in order to avoid the process of VK_2_ degradation.

### Preparation of the Caco-2 cell culture for voltammetric measurements and analysis of vitamin K_2_-MK4 ability of eukaryotic cell membrane penetration

When examining the effect of various VK_2_ homologs on eukaryotic cells, it is very important to try to answer the question whether in the large intestine, i.e. in the place where the bacterial flora synthesizes vitamin K_2_, this vitamin can penetrate into the intestinal epithelial cells despite the lack of bile and pancreatic enzymes. We considered the answer to this question crucial from the point of view of the legitimacy of targeted VK_2_ supplementation through its natural producers, i.e. selected bacterial strains. In order to test the penetration capacity of VK_2_ through the membrane, the Caco-2 cells were cultivated with synthetic K_2_-MK4 in a two concentration levels: 4.4 and 44 mg/L.

After 24, 48 and 72 h of the experiment, VK_2_ was quantified both inside (crushed Caco-2 cells) and outside of cells in the post-culture fluids (post-culture DMEM medium and PBS from a third wash of Caco-2 cells). The preparation of the collected samples for voltammetric analysis were as follow:

- Post-culture DMEM medium was analyzed without preliminary preparation.
- To the Caco-2 cells left after collecting supernatant, PBS was added to remove VK_2_-MK4 adsorbed on the cell surface. The PBS solution was added in a strong stream causing the tissue to detach from the culture plate and ensuring complete cleanse of the tissue from the vitamin K_2_-MK4 leftovers (this operation was repeated three times).

PBS left from third rinsing of the Caco-2 cells was directly taken for the analysis, without any treatment.

c. To the separated Caco-2 cells remained after PBS rinsing, 200 µL of pure PBS was added. Such prepared sample was shaken on the ultrasonic shaker for 30 min in order to destroy the cell membrane and get access to VK_2_ that might be contained inside the cell. In the next step, cells with PBS were centrifuged for 5 min at the speed of 2000 rpm. The obtained supernatant was then analyzed voltammetrically.

Both samples, with and without K_2_-MK4 addition, were prepared using the same procedure, which was unchanged for different time of cell culture incubation. Voltammetric analysis was performed according to the procedure described in point 2.3. As far as possible, all activities and measurements were performed without exposure of the samples to light.

### Statistical analysis

Statistical analysis was performed using OriginLab 2021b. Obtained data were tested with t-Student Test and presented as mean ± SD of at least three measurement replicates without outliers. On the graphs two levels of significance was presented, *p*-values < 0.05 marked as a *, and *p*-values < 0.01 marked as a **.

## Results

### Bacterial production of vitamin K_2_

In order to pinpoint the most efficacious vitamin K_2_ producers, we examined different species and bacterial strains (86 in total) under identical passage conditions, including both etiological agent bacteria and GRAS (Generally Recognized As Safe) status bacterial strains. Detailed culture procedures, sample preparation, and analysis are described in Sect. 2.1, 2.2, and 2.3, respectively. The lowest and the highest total VK_2_ content (sum of homologs) concentration values measured in the post-culture supernatants for every bacteria strains are both outlined in Table 1.

**Table 1:** Bacterial producents of vitamin K_2_. c_min_ and c_max_ refer to the lowest and the highest concentration of VK_2_ measured in supernatant obtained within a 4-day long measurement cycle. Each strain was tested in triplicate

Bacterial growth conditions	Bacteria species		Number of tested strains, N	c_min_, mg/L	c_max_, mg/L
Strictly anaerobic	*Clostridium*	*perfringens*	N = 3	0.03	0.79
		*difficile*	N = 1	<LOD	<LOD
	*Bacteroides*	*stercoris*	N = 1	<LOD	0.07
		*vulgatus*	N = 2	0.03	0.05
Microaerophilic	*Lactobacillus*	*plantarum*	N = 7	0.49	4.6
		*rhamnosus*	N = 7	0.36	2.5
		*acidophilius*	N = 7	0.18	0.69
		*gasseri*	N = 5	0.04	0.37
	*Bifidobacterium*	*dentium*	N = 3	0.11	1.2
		*longum*	N = 1	0.61	3.6
		*animalis*	N = 1	<LOD	0.07
		*pseudocatenulatum*	N = 1	0.07	0.17
		*adolescentis*	N = 1	0.07	0.11
Aerobic	*Escherichia*	*coli*	N = 14	0.21	1.2
	*Klebsiella*	*pneumoniae*	N = 3	0.13	0.28
		*oxytoca*	N = 2	<LOD	<LOD
	*Enterobacter*	*cloacae*	N = 7	<LOD	<LOD
	*Hafnia*	*alvei*	N = 1	<LOD	<LOD
	*Pseudomonas*	*aeruginosa*	N = 1	<LOD	<LOD
	*Staphylococcus*	*aureus*	N = 7	<LOD	<LOD
		*haemolyticus*	N = 1	0.01	0.13
		*epidermidis*	N = 1	0.06	0.32
	*Enterococcus*	*fecalis/faecium*	N = 8	0.16	1.4
	*Bacillus*	*subtilis* Natto *(control strain)*	N = 1	0.22	1.8
** N total = 86**					

<LOD – concentration of VK_2_ below the limit of detection of the DP AdSV method

Both anaerobic and aerobic bacterial species representatives produce VK_2_. However, the anaerobic and microaerophilic species performed significantly better (up to 4.6 mg/L of VK_2_) than aerobic species (up to 1.8 mg/L of VK_2_). The species diversity was also evident, as reflected by VK_2_ concentration levels for different genus, e.g., *Lactobacillus*. The highest VK_2_ production level was observed for the *L. plantarum* species (max. 4.6 mg/L), whereas the lowest VK_2_ concentration was measured for the *L. gasseri* species (min. 0.04 mg/L). A few strains from *L. rhamnosus* and *L. acidophilus* species did not produce any VK_2_. Some *Bifidobacterium* strains, along with the *Lactobacillus* species, are the principal human gastrointestinal tract inhabitants and turned out to be excellent VK_2_ producers, reaching a maximum 3.6 mg/L concentration for the *Bifidobacterium longum*. For the aerobic species, the most prominent outcome was observed for *Bacillus subtillis Natto* (about 1.8 mg/L). *Escherichia coli*, one of the most commonly found aerobic bacteria in the mammalian lower intestine, also exhibited VK_2_ synthesis capability. Among 14 tested *E.coli* strains, four of them did not produce measurable VK_2_ amounts. In other strains, the maximum measured VK_2_ concentration was about 1.2 mg/L, with a minimum of 0.12 mg/L. Yet, the maximum VK_2_ amount produced by *E. coli* was twice as low as the highest achieved *Lactobacillus* genus concentration and even lower than the *Bacillus* and *Bifidobacterium* results.

For some other aerobic genera, such as *Klebsiella oxytoca*, *Enterobacter cloacae*, *Hafnia alvei*, *Pseudomonas aeruginosa*, and *Staphylococcus aureus*, no VK_2_ production was observed.

### Vitamin K_2_-MK4 cell interior penetration and water media solubility

In order to investigate the ability of VK_2_ to penetrate the Caco-2 tissue cell membrane, the appropriate studies on Caco-2 cells with schort-chain VK_2_-MK4 homolog addition were performed. Procedure details are described in Sect. 2.5, and the test results are compiled in Table 2. Culturing Caco-2 cells with synthetic K_2_-MK4 at two concentrations (4.4 and 44 mg/L) was performed to test VK_2_ cell membrane permeability. The first value corresponds to typical VK_2_ concentrations produced by some strains of Lactobacillus plantarum (Table 1). However, we decided to increase this concentration tenfold (44 mg) to determine whether such vitamin K_2_-MK4 concentration can penetrate the membranes of intestinal epithelial cells.

**Table 2:** Vitamin VK_2_-MK4 penetration inside the Caco-2 tissue. Measured vitamin VK_2_-MK4 levels in mg/L

Sample	Without VK_2_-MK4 addition			4.4 mg/L VK_2_-MK4 addition			44 mg/L VK_2_-MK4 addition		
	24 h	48 h	72 h	24 h	48 h	72 h	24 h	48 h	72 h
**Post-culture supernatant (DMEM)**	<LOD	<LOD	<LOD	2.3	1.3	2.8	13	8.6	9.0
**PBS form third rinsing**	<LOD	<LOD	<LOD	0.2	0.1	0.2	0.6	0.6	0.7
**Crushed Caco-2 cells**	**<LOD**	**<LOD**	**<LOD**	**0.2**	**0.1**	**1.7**	**12**	**22**	**11**

In the Caco-2 tissue controls (with no VK_2_-MK4 contact), VK_2_ was not detected; in tissues subsidized with VK_2_-MK4, VK_2_ could be measured in all samples. Namely, for Caco-2 tissue samples (4.4 mg/L of VK_2_-MK4 addition), the highest VK_2_-MK4 concentration was observed in the DMEM samples; the maximum of 2.8 mg/L was reached after 72 h of tissue contact with VK_2_-MK4 (64% of the initial concentration). Results were comparable in the crushed Caco-2 cells - maximum VK_2_-MK4 concentration was observed after 72 h of exposure (39% of the initial VK_2_-MK4 concentration); it was again measurable in 24- and 48-hour samples (4.5% and 2.3% of starting concentration respectively).

In Caco-2 tissues that contacted the 44 mg/L VK_2_-MK4 solutions, significantly higher VK_2_ contents were detected. The highest 22 mg/L VK_2_-MK4 concentrations were observed in crushed Caco-2 cells after a 48-hour period (50% of initial VK_2_-MK4 concentration). Conversely, tissues that contacted the VK_2_-MK4 solution for 24 and 72 h revealed lower VK_2_ levels of about 12 and 11 mg/L (27% and 25% of initial VK_2_-MK4 concentrations).

To recapitulate, with a low concentration of 4.4 mg/L VK_2_-MK4, VK_2_ concentration in the Caco-2 tissue systematically increased by 0.1, 0.2, and 1.7 mg/L after 24, 48, and 72 h. With a high concentration of 44 mg/L VK_2_-MK4, VK_2_ levels in tissue initially grew to 12 mg/L after 24 h, then 22 mg/L after 48 h, and then suddenly decreased to 11 mg/L after 72 h, respectively. It was significantly higher than the VK_2_ concentration determined in PBS form third rinsing, confirming that VK_2_-MK4 can penetrate the Caco-2 tissue cell membrane. Similar outcomes were accomplished after the experiment was repeated. At this phase of the study, the reason for the initial increase and the subsequent decrease in VK_2_ tissue concentration after being exposed to 44 mg/L VK_2_-MK4 solutions is impossible to justify.

### Influence of vitamin K_2_ homologs on Caco-2 cell apoptosis and necrosis

Three synthetic VK_2_ homologs (VK_2_-MK4, VK_2_-MK7 and VK_2_-MK9), DMEM (10% FBS), 2 µM staurosporine, 2.0 mM H_2_O_2_ solution, and the *L. plantarum* cultivation supernatant (natural VK_2_ producer) were utilized to examine their impact on the Caco-2 intestinal epithelial cell line’s necrosis and apoptosis. The experiment process is described in Sect. 2.4.1, while the test outcomes are compiled in Table 3.

**Table 3:** Effect of synthetic VK_2_ homologs in selected concentrations, DMEM (10% FBS), staurosporin solution, hydrogen peroxide and supernatant from the cultures of *L. plantarum* strains on the apoptosis and necrosis of cells of the intestinal epithelial line Caco-2

Sample	VK_2_ concentration, mg/L (µM)	Living cells, %	Necrotic cells, %	Apoptic cells, %
VK_2_-MK4 (synthetic VK_2_ homologe)	1.1 (2.5)	93	0	7
	2.2 (5)	85	6	9
	4.4 (10)	88	0	12
	6.7 (15)	86	8	6
	8.9 (20)	87	0	13
	11 (25)	94	0	6
VK_2_-MK7 (synthetic VK_2_ homologe)	1.6 (2.5)	88	0	12
	3.2 (5)	90	6	4
	6.5 (10)	82	3	15
	9.7 (15)	95	0	5
	13 (20)	87	0	13
	16 (25)	89	0	11
VK_2_-MK9 (synthetic VK_2_ homologe)	2.0 (2.5)	97	1	2
	3.9 (5)	98	0	2
	7.9 (10)	97	0	3
	12 (15)	96	3	1
	16 (20)	93	1	6
	20 (25)	96	0	4
DMEM (10% FBS) (negative apoptosis and necrosis control)	0	94	3	3
Staurosporin (concentration of 2 µM – positive apoptosis control)	0	43	19	38
H_2_O_2_ (concentration of 2.0 mM – positive necrosis control)	0	66	27	7
* L. plantarum* (natural vitamin K_2_ producent) supernatant	0	88	7	5

VK_2_ homolog concentrations ranging from 2.5 to 25 µM used in the experiment were selected according to VK_2_ bacterial production results, where the highest obtained result was about 4.4 mg/L (Table 1). Based on Table 3, we concluded that none of the VK_2_ homologs (even highly concentrated) significantly affected the necrotic processes of the intestinal epithelial line Caco-2. VK_2_-MK4 and VK_2_-MK7 homologs slightly affected the apoptotic processes, but finding a specific vitamin concentration association may not be easy. Vitamin VK_2_-MK9 (regardless of concentration) does not affect apoptosis and necrosis of Caco-2 cells whereas *L. plantarum* culture supernatant showed little impact.

### Impact of vitamin K_2_ homologs on cytokines

**Fig. 1:**
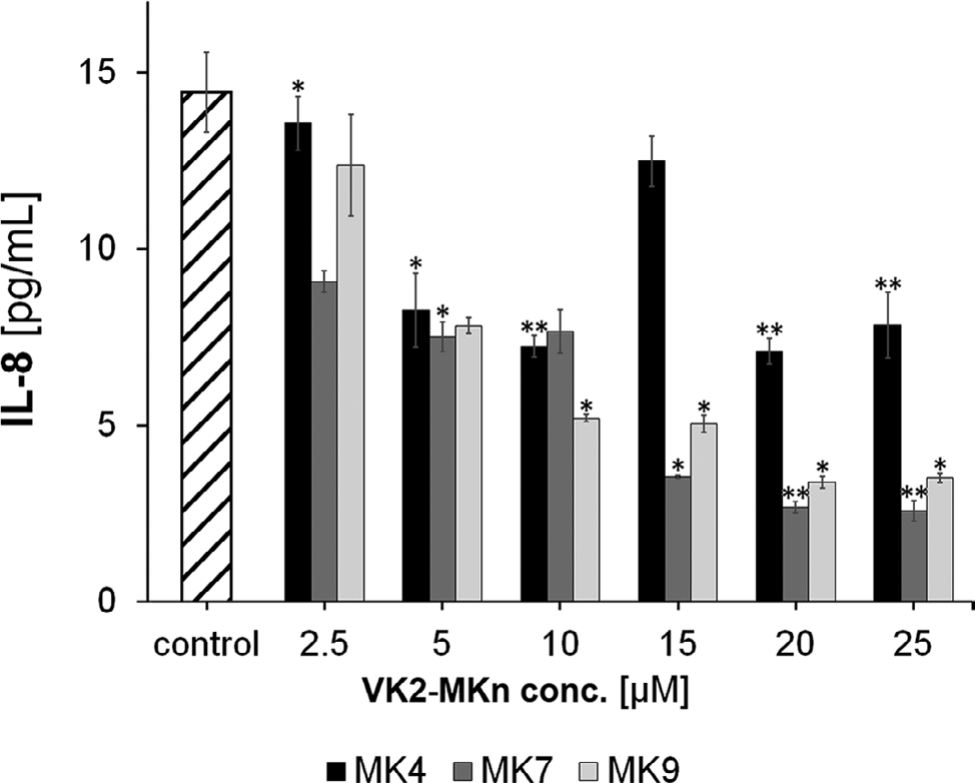
The impact of VK_2_ homologs (VK_2_-MK4, VK_2_-MK7 and VK_2_-MK9) and their concentration on the level of pro-inflammatory IL-8 in the cells of the Caco-2 intestinal epithelial cell line (incubation time 24 h). Changes in the cytokine levels was measured with respect to control sample (Caco-2 with culture medium, without VK_2_). * p < 0.05 ** p < 0.01

The following research stage involved testing the influence of VK_2_ homologs (VK_2_-MK4, VK_2_-MK7, VK_2_-MK9) on cytokine secretion in Caco-2 cells. VK_2_ homolog concentration was examined in the 0 to 25 µM range, with a 24-hour incubation time. The experiment process is described in Sect. 2.4.2. Test results are presented in Fig. 1. Cytokine level changes were measured regarding the control sample (Caco-2 with culture medium, without VK_2_).

As shown in Fig. 1, each VK_2_ homolog causes a significant decrease in proinflammatory IL-8 concentration in Caco-2 human colorectal adenocarcinoma cells. Although this decrease becomes more significant as VK_2_ concentration rises, the most remarkable IL-8 changes are observed above 15 µM VK_2_ for VK_2_-MK7, and VK_2_-MK9 homologs.

The statistical significance of the obtained outcomes was tested on two levels and presented in Fig. 1. Unfortunately, based on the performed tests, there was no measurable signal of other tested cytokines, such as IL-6, IL-10, and TNFα (both in the control tissue and the tissue with synthetic VK_2_: VK_2_-MK4, VK_2_-MK7, and VK_2_-MK9 homolog addition). Therefore, these examinations implicate that vitamin K_2_ in any tested form did not stimulate cytokine secretion detected in the Caco-2 cell line.

**Fig. 2:**
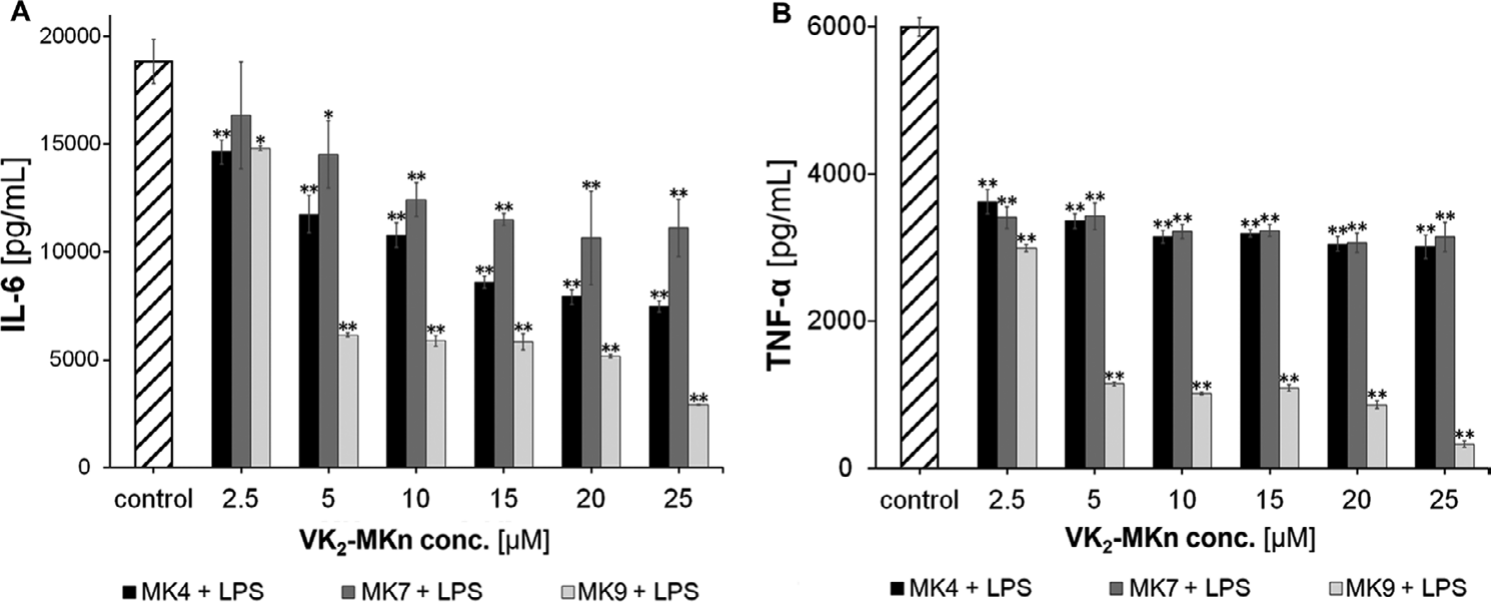
The impact of VK_2_ homologs (VK_2_-MK4, VK_2_-MK7 and VK_2_-MK9) and their concentration on the secretion of pro-inflammatory cytokines: IL-6 with addition of 100 ng/mL of LPS (A) and TNFα with addition of 100 ng/mL of LPS (B) in the RAW 264.7 mouse macrophage cell line (incubation time, 24 h). Changes in the cytokine levels was measured with respect to control sample (RAW 264.7 cells with culture medium stimulated with LPS, without VK_2_). Statistical significance was evaluated in comparison with RAW 264.7 cells with addition of 100 ng/mL of LPS (B and D). * p < 0.05 ** p < 0.01

We likewise investigated the impact of the VK_2_ homologs (VK_2_-MK4, VK_2_-MK7, and VK_2_-MK9) on the proinflammatory cytokines in the mouse RAW 264.7 cells. The cell culture preparation process, experiment background, and sample preparation are presented in point 2.4.2, and the measurement results are compiled in Fig. 2. Cytokine level shifts were examined corresponding to the control sample (RAW 264.7 with culture medium and RAW 264.7 with culture medium stimulated by LPS, both without VK_2_). The statistical significance of the obtained outcomes was tested on two levels and is illustrated in Fig. 2.

Data analysis indicates that each of the VK_2_ homologs caused a significant decrease in the proinflammatory IL-6 and TNFα concentration in the RAW 264.7 mouse macrophage cell line. It was observed that the long-chain VK_2_-MK9 homolog effectively reduced proinflammatory cytokine levels.

This activity was dose-dependent because the greatest decrease in IL-6 concentration level (versus control of 18,829 pg/ml) was observed for VK_2_ concentrations starting from the value of 5 µM, which is the value similar to the VK_2_ concentration produced by microaerophilic bacterial strains (Table 1). The highest decrease was observed for 25 µM VK_2_-MK9 concentration; they were respectively 67% (6144 pg/ml) for 5 µM and 85% (2905 pg/ml) for 25 µM. Similar situation was obtained for the investigation of TNFα behavior (control value of 5995 pg/ml). A large decrease in the TNFα was observed from the VK_2_ concentration of 5 µM (81% decrease versus control (1149 pg/ml)), and the lowest values were obtained for 25 µM VK_2_ concentration (95% decrease versus control (327 pg/ml)).

## Discussion

Colorectal cancer (CRC) is a complex multifactorial digestive disease and the third most common cause of cancer-related mortality worldwide. According to oncologists, gastroenterologists, and dieticians, a proper diet (enriched with calcium, selenium, or vitamin D_3_) is considered a CRC prevention measure. Studies on the impact of a diet rich in fermented soy products (Natto, miso) containing high vitamin K_2_ concentration provide promising outcomes. Clinical studies indicate that VK_2_ supplementation (a daily dose of 45 mg) may lower the risk of primary liver cancer by 80% (compared to the control group). A cohort study conducted in Heidelberg (11,928 men aged 40–69) confirmed the strong impact of VK_2_ on prostate cancer development reduction.

Moreover, Amalia et al. demonstrated a unique role of VK_2_ supplementation in inhibiting the growth of radiation therapy-resistant cancer cells. However, oral VK_2_ supplementation can be inefficient, as it reacts with stomach acid, bile salts, and pancreatic enzymes in the human digestive system, which leads to significant vitamin losses. Hence, even a high VK_2_ dose administered orally does not ensure sufficient vitamin absorption.

Vitamin K_2_ is a bacterially derived compound that can be supplied to the body exogenously and endogenously. Exogenous vitamin VK_2_ is produced beyond the human body and is delivered through fermented products or dietary supplements containing synthetic equivalents of the vitamin. Foods rich in VK_2_ are mainly long-maturing cheeses, dairy products, and fermented soybeans, including Nattō and miso. These fermentation processes are usually carried out by animally-derived and plant-derived bacterial genus (*Lactococcus, Streptococcus, Leuconostoc*, and *Bacillus*).

Human gut microbiota produces endogenous VK_2_. The appropriate qualitative and quantitative composition of the intestinal microbiota affects the vital functions of the host. However, acute and chronic enteritis, antibiotic therapy, or chemotherapy can lead to radical microbiological imbalance causing a significant general deficiency of vitamins B and K_2_ in our body [40, 41]. Hence, to effectively supplement endogenous VK_2_ through the targeted probiotic flora administration, it is essential to indicate which bacterial strains belonging to the human intestinal microbiota are its most prominent producers. The literature indicates that VK_2_ MK4 to MK9 homologs are synthesized by the majority of saprophytic bacteria that colonize the human gastrointestinal tract [42]. However, our research shows that the most notable VK_2_ producers are microaerophilic and anaerobic bacteria (*Lactobacillus, Bifidobacterium* and *Bacillus*). In particular, *Lactobacillus plantarum* species produce the highest VK_2_ amounts comparable to the VK_2_ production typical for the *Bacillus subtilis* strain in Natto. However, plant-derived *Natto Bacillus subtilis* shows a limited adherence to human intestinal epithelial cells and mucuse layer. In addition, for most consumers, all *Bacillus*-fermented foods look, smell, and feel unappetizing (they are tolerable only to Japanese, Chinese, and Korean citizens).

Our hypothesis states that oral supplementation of VK_2_ positive probiotic bacteria (*Lactobacillus* and *Bifidobacterium*) is more reasonable than choosing *Bacillus subtilis* or synthetic VK_2_. Firstly, the suggested genera are typical components of healthy human microbiota demonstrating a solid affinity to the intestinal epithelium, ensuring long-term and effective gastrointestinal tract colonization. Secondly, *Lactobacillus* and *Bifidobacterium* (possessing probiotic properties) can also regulate the intestinal barrier’s tightness, inhibit intestinal pathogen proliferation, produce antioxidant enzymes (e.g. catalase), and increase anti-inflammatory cytokine secretion. In addition, products fermented by these probiotic bacteria taste and smell well.

There are very few clinical studies in the literature that investigate bacterially-produced VK_2_. Zhang hypothesized that *Lactobacillus casei* and vitamin K_2_ could benefit patients with colon cancer by modulating adiponectin. Another study found that *Lactobacillus* fermented yogurt can increase the absorption of VK_2_-MK7 in humans, suggesting synergistic effects of *Lactobacillus* and VK_2_-MK7 administered orally. So far, *Lactobacillus* bacteria have been practically applied to prevent and treat intestinal infections, reproductive organ diseases, and autoimmune disorders, including food and skin allergies. However, scientists deliver many conflicting beliefs on the absorption effectiveness of endogenous VK_2_ naturally produced by the human microbiome in the digestive tract. The prevailing opinion among scientists is that only exogenous VK_2_ supplementation is reasonable due to its fat-solubility, which allows it to be absorbed mainly in the duodenum and small intestine. They also state that VK_2_ produced in the large intestine is marginally significant and should be ignored in the overall vitamin balance.

Therefore, in our publication, we tried to answer the fundamental question whether VK_2_ is solely fat-soluble? From our preliminary in vitro studies (using the Caco-2 line and the selected VK_2_-MK4 homolog), we presented (Sect. 3.2) that synthetic VK_2_-MK4 dissolved in distilled water at a concentration of 4.4 mg/L and is well absorbed by Caco-2 cells. After a 72-hour experiment, its estimated cell concentration was 1.7 mg/L (30% of the initial vitamin concentration in its aqueous solution). These results supported our hypothesis that vitamin K_2_ does not require to be suspended in fats to effectively penetrate the intestinal epithelial cells. Y. Yanagisawa and H. Sumi’s teams made similar observations [43, 44]. They found that vitamin K produced by *Bacillus subtilis Natto* becomes water-soluble through the formation of an intracellular complex with the protein and is released to the extracellular space during bacterial cell proliferation. Moreover, the same scientists have shown that water-soluble VK_2_ is much more stable in the blood and maintains a high VK_2_ concentration. Thus, we hypothesize that VK_2_ produced by bacteria in the colon can penetrate directly into the intestinal epithelial cells and absorb without the presence of fatty environment and the involvement of pancreatic enzymes and bile salts (both in the upper and lower gastrointestinal tract).

This twofold VK_2_ supply increases the total concentration of this vitamin and its homologs in our body which can probably perform additional biological functions.

We wonder why gut bacteria produce VK_2_, and if they use it only for their own needs or share it with the host cells. We strive to discover whether the individual VK_2_ homolog profile is synthesized depending on bacteria genus and species and if individual homologs have different biological functions. The number of questions about bacterial VK_2_ is constantly increasing. Some of these questions have already been answered. Bacteria can use VK_2_ in the electron transport chain for cellular respiration, and this plays a vital role in the system that converts ADP (adenosine diphosphate) energy to ATP (adenosine triphosphate) [45–47]. Electron donors, under the influence of appropriate enzymes, transfer 2 electrons to VK_2_, and then, using another enzyme, they are transferred from VK_2_ to the electron acceptor [48]. This menaquinone-based energy production method is effective for both Gram-positive and Gram-negative bacteria. It participates in bacteria photosynthesis and nitrogen fixation regulation [49].

VK_2_ significantly impacts human cell biological functions - it promotes blood coagulation, bone fracture healing, and osteogenesis. It plays a vital role in regulating matrix GLA protein and osteocalcin activity during extrahepatic tissue transportation [42].

**Fig. 3:**
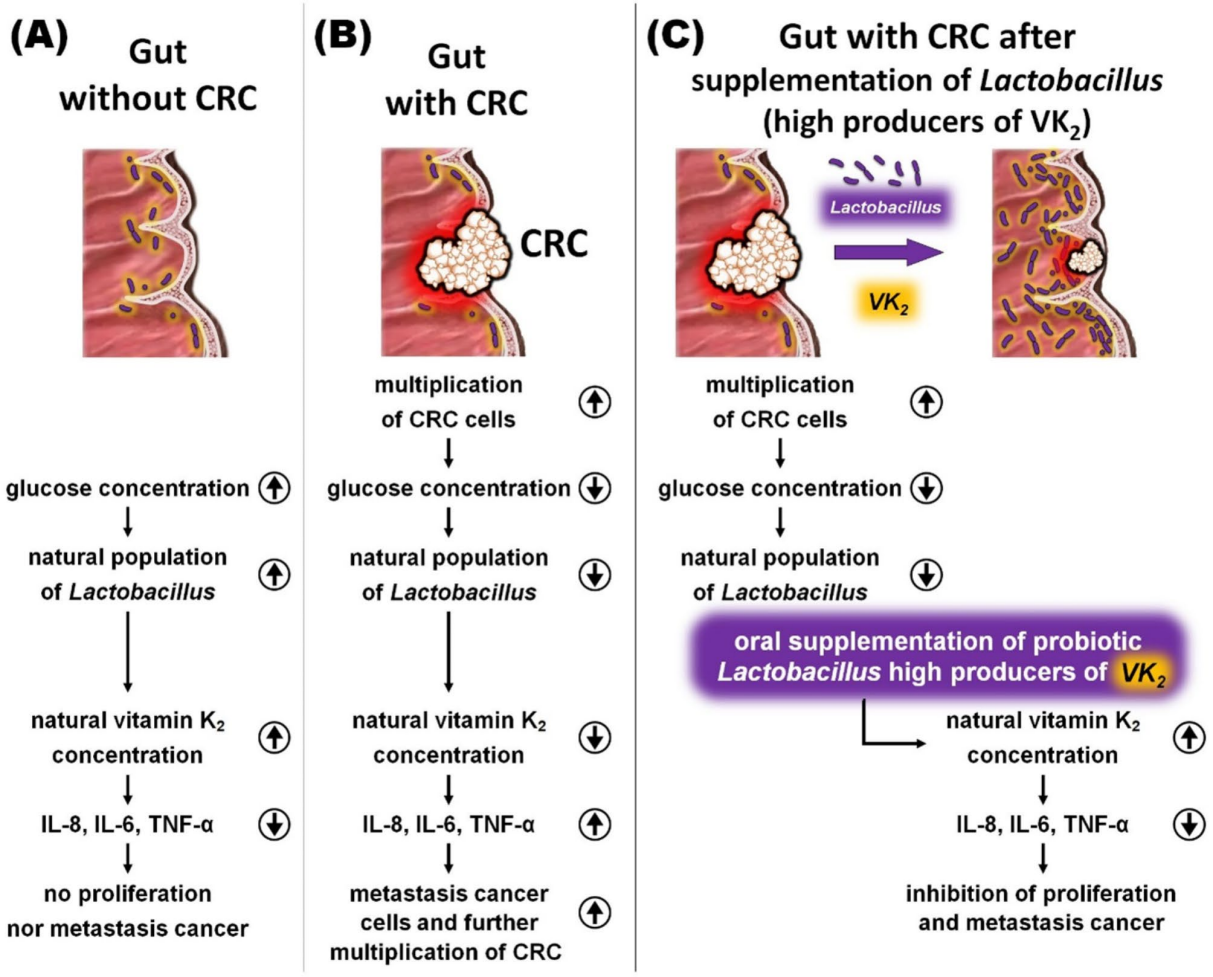
The proposed scheme of possible vitamin K_2_ impact on the gut with and without colorectal cancer

Our in vitro studies showed that all synthetic VK_2_ homologs, including VK_2_-MK4, VK_2_-MK7 and VK_2_-MK9, did not significantly affect the apoptosis and necrosis of Caco-2 cells. It was confirmed by comparing the results with a series of control samples. However, VK_2_ homologs (especially the longer chain VK_2_-MK7 and VK_2_-MK9) significantly reduced pro-inflammatory cytokine Interleukin-8 (IL-8) secretion by Caco-2 cells. IL-8 is a pro-inflammatory chemokine that has been associated with neutrophil chemotaxis induction and degranulation. Lee et al. report that IL-8 regulates the proliferation, migration, and angiogenesis of a colon cancer cell line [50]. The cytokine inhibition level depended on VK_2_ concentration and its homolog type. The most significant 80% decrease in IL-8 was observed for VK_2_-MK7 (20–25 µM concentration) compared to the Caco-2 culture without the addition of the tested VK_2_ homologs. The same team showed IL-8 levels in patients with a more advanced form of colorectal cancer were ten times higher than in asymptomatic patients. Therefore, IL-8 level reduction in the gastrointestinal tract is highly desirable in patients with chronic diseases, including CRC. Recent studies have shown IL-8 overexpression in various tumor environments, including colon and lung cancer. The proposed scheme of the possible effects of the VK_2_ supplementation on the gut with and without colonrectal cancer is presented in Fig. 3.

In our view, in the future, bacterial production of long VK_2_ homologs may become a natural method of inhibiting IL-8 secretion by colon cancer cells. Significantly, bacterially produced VK_2_ stays on the surface of intestinal cells and can be absorbed by these cells immediately and efficiently. Unfortunately, only probiotic bacteria from the intestinal microbiota (e.g., *Lactobacillus*) can be considered, as they adhere well to intestinal mucosa cells for an extended period. In contrast, the *Bacillus subtilis Natto* bacteria constitute only the planktonic microorganism, which quickly leaves the intestine and is excreted with the feces from the body.

By examining the impact of synthetic VK_2_ homologs on the LPS-stimulated murine macrophage (RAW 264.7 line) pro-inflammatory cytokines secretion (IL-6, TNFα), we observed a significant decrease in both cytokines under the influence of K_2_-MK9 homolog (5 to 25 µM concentration). Compared to the control group, the reduction was 70–80% for IL-6 and over 95% for TNFα. Interestingly, the remaining homologs (K_2_-MK4 and K_2_-MK7) also showed an inhibitory impact on the secretion of pro-inflammatory cytokines, but it was almost two times lower than that of K_2_-KM9.

Notably, our research results agree with many study groups examining the subject. VK_2_ inhibits the cytokine storm, mainly through significant pro-inflammatory cytokine inhibition, which may reduce tumor growth and metastases. Maihofner et al. compared IL-6 and TNFα levels (both in the serum and the tumor) in colorectal cancer patients and healthy people [51]. It turned out that the cytokine level in cancer patients was significantly higher. These observations imply that IL-6 and TNFα can play an essential role in cancer pathogenesis by modulating the expression of IL-8 receptors. Schneider et al. demonstrated a correlation between IL-6 levels and the growth rate of epithelial colon cancer cells and tumor relapse [52]. Based on literature data related to colon cancer, the secretion of IL-8 dramatically increases during the process of metastasis [51]. High level of IL-8 is a bad prognosis for further treatment [53–55]. Thus, all factors (including some species of bacteria colonizing the GI tract) reducing IL-8 indirectly contribute to the reduction of metastasis.

Observations made by the researchers mentioned above prompted us to search for natural methods of pro-inflammatory cytokine secretion inhibition (IL-6, TNFα, and IL-8) in the intestinal tumor environment. We want to suggest oral probiotic bacteria supplementation, which releases long-chain VK_2_ homologs into the extracellular space.

Based on our research, the bacterial production of VK_2_ may depend not only on a specific genus (e.g., *Lactobacillus, Bifidobacterium*) but may even be a strain-dependent feature.

However, we still do not know whether the short VK_2_ homologs (such as K_2_-MK4) or the long side chains (K_2_-MK7, K_2_-MK9) are more effective in inflammation inhibition. Some authors believe that oral VK_2_ supplementation of K_2_-MK4 (shorter isoprenoid chains) reduces overall inflammation better than K_2_-MK7 (longer isoprenoid chains) [56]. Nevertheless, other studies using endothelial cells showed the opposite, i.e., K_2_-MK7 was more potent than K_2_-MK4 [57].

Therefore, the next stage of our research will be to conduct in vivo tests on a mouse model of colorectal cancer with simultaneous supplementation with probiotic bacteria characterized by high production of vitamin K_2_ with long homologs (K_2_-MK7 and/or K_2_-MK9). As part of further research the selected strains will be characterized in terms of their probiotic properties, such as regulating intestinal barrier integrity, inhibiting the multiplication of intestinal pathogens, production of antioxidant enzymes and increasing the secretion of anti-inflammatory cytokines.

## Conclusion

As a result of examining 86 bacterial strains of intestinal origin (incubated for 24, 48, 72, and 96 h), we demonstrated that their common feature is vitamin K_2_ production. The highest VK_2_ concentration measured in the bacterial supernatant sample was found for the following genus: *Lactobacillus* (up to 4.6 mg/L (10 µM)), *Bifidobacterium* (up to 3.6 mg/L (8 µM)), and *Bacillus* (up to 1.8 mg/L (4 µM). The amount of vitamin K_2_ produced depends on a specific species and a strain. Basing on the obtained results, we chose one of the best VK_2_ producers from *Lactobacillus* genus and applied it to the future in vivo research.

Our research presented the possibility of VK_2_ penetration into the Caco-2 cell interior and water-solubility (some VK_2_ forms), which is the crucial behavior for the possibility of inhibition the pro inflammatory cytokines.

The in vitro studies on the human colon adenocarcinoma Caco-2 cell line showed that all tested VK_2_ homologs (especially long-chain) had a high potential to inhibit IL-8 secretion. In addition, all tested VK_2_ homologs retained the ability to the inhibit secretion of the pro-inflammatory cytokines (IL-6 and TNFα in the mouse RAW 264.7 cells). The long-chain MK9 homolog appeared to be the most effective.

The obtained results demonstrate that VK_2_ produced by intestinal bacteria can contribute to the inhibition of pro-inflammatory cytokines reducing colorectal cancer growth and metastasis.

## Data Availability

The data that support the findings of this study are available from the corresponding author upon reasonable request.

## Abbreviations

ADP: Adenosine Diphosphate
ATP: Adenosine Triphosphate
Caco-2: human intestinal epithelial cells
CGMDE: Controlled Growth Mercury Drop Electrode
CRC: Colorectal Cancer
dE: pulse amplitude (voltametric parameter)
DMEM: Dulbecco’s Modified Eagle Medium
DP AdSV: Differential Pulse Adsorptive Stripping Voltammetry
E_acc_: accumulation potential (voltametric parameter)
E_s_: potential step (voltametric parameter)
FBS: Fetal Bovine Serum
GRAS: Generally Recognized As Safe, bacterial strains status
IL-6: Interleukin 6
IL-8: Interleukin 8
IL-10: Interleukin 10
LOD: Limit Of Detection
LPS: Lipopolysaccharide
PBS: Phosphate-Buffered Saline Solution
PI: Propidium Iodide
RAW: mouse macrophage cells
t_acc_: accumulation time (voltametric parameter)
t_imp_: pulse period (voltametric parameter)
TNFα: Tumor Necrosis Factor α
VK_2_: Vitamin K_2_, menaquinone
VK_2_-MKn: Vitamin K_2_ homologe with n isoprenyl units in their side chain
